# Importance of interdisciplinarity in modern oncology: results of a national intergroup survey of the Young Oncologists United (YOU)

**DOI:** 10.1007/s00432-023-04937-2

**Published:** 2023-06-01

**Authors:** Matthias Mäurer, Jonas Staudacher, Robert Meyer, Irina Mäurer, Lazaros Lazaridis, Michael Müther, Tobias Huber, Nils P. Sommer, Daniel F. Fleischmann, Lukas Käsmann, Sonia Ziegler, Cornelia Kropf-Sanchen, Julia Wikert, Klaus Pietzner, Adrien Holzgreve, Tim Nestler, Carolin Siech, Max-Johann Sturm, Sabrina Sulzer, Kathrin Heinrich, Arndt Stahler, on behalf of the Young Oncologists United (YOU)

**Affiliations:** 1 Department of Radiotherapy and Radiation Oncology, University Hospital Jena, Jena, Germany; 2 Department of Gastroenterology, Rheumatology and Infectiology, Charité Universitätsmedizin Berlin, Campus Benjamin Franklin, Berlin, Germany; 3 Institute of Human Genetics and Genomic Medicine, University Hospital Aachen, Medical Faculty, RWTH Aachen University, Aachen, Germany; 4 Department of Hematology, Oncology, Hemostaseology and Stem Cell Transplantation, University Hospital Aachen, Faculty of Medicine, RWTH Aachen University, Aachen, Germany; 5 Department of Neurology, Neurooncology Center, University Hospital Jena, Jena, Germany; 6 Department of Neurology and Center for Translational Neuro- and Behavioral Sciences (C-TNBS), Division of Clinical Neurooncology, University Medicine Essen, University Duisburg-Essen, Essen, Germany; 7 Department of Neurosurgery, University Hospital Münster, Münster, Germany; 8 Department of General, Visceral and Transplant Surgery, University Medical Center Mainz, Mainz, Germany; 9 Department of General, Visceral, Thoracic and Vascular Surgery, University Hospital Bonn, Bonn, Germany; 10 Clinic and Polyclinic for Radiotherapy and Radiooncology, LMU Clinic Munich, LMU Munich, Munich, Germany; 11 Clinic and Polyclinic for Radiation Therapy and Radiooncology, University Medical Center Göttingen, Göttingen, Germany; 12 Department of Internal Medicine II, University Hospital Ulm, Ulm, Germany; 13 Clinic and Polyclinic for Palliative Medicine, LMU Klinikum München, Munich, Germany; 14 Department of Gynecology, Charité Universitätsmedizin Berlin, Berlin, Germany; 15 Clinic and Polyclinic for Nuclear Medicine, University Hospital Munich, LMU Munich, Munich, Germany; 16 Clinic for Urology, Bundeswehr Central Hospital Koblenz, Koblenz, Germany; 17 Department of Urology, University Hospital Frankfurt, Frankfurt am Main, Germany; 18 Department of Gastroenterology, Gastrointestinal Oncology and Endocrinology, University Medical Center Göttingen, Göttingen, Germany; 19 Medical Clinic and Polyclinic III, University Hospital Munich, LMU Munich, Munich, Germany; 20 Charité University Medicine, Medical Clinic m. S. Hematology, Oncology and Tumor Immunology, Berlin, Germany; 21 Berlin Institute of Health at Charité - Universitätsmedizin Berlin, Berlin, Germany; 22 Center for Integrated Oncology Aachen Bonn Cologne Duesseldorf (CIO ABCD), Bonn, Germany

**Keywords:** Interdisciplinary networks, Junior scientists

## Abstract

**Purpose:**

Modern, personalized treatment concepts in oncology require an interdisciplinary and multiprofessional collaboration. In addition to its relevance in patient care, interdisciplinary collaboration is also becoming increasingly important in clinical research as well as medical education and resident training in oncology.

**Methods:**

Between November 2021 and March 2022, an online survey was conducted among German early career research groups, represented by Young Oncologists United (YOU). The aim was to identify the status and need for interdisciplinarity at clinic, educational, and research levels.

**Results:**

A total of 294 participants completed the questionnaire in full. 90.7% of the respondents fully or predominantly agreed with the statement that interdisciplinary work plays a major role in their daily clinical work. 78.9% wished for more interdisciplinary collaboration. Of the 49.7% of participants who have never participated in an interdisciplinary research project, 80.1% said they would like to participate in such a study project in the future. Lack of time resources, too much organizational effort, and possible political conflicts between institutions were identified as factors that make practical implementation difficult. 74.1% declared their willingness to become active in an oncology early career research group.

**Conclusion:**

Interdisciplinary collaboration has become increasingly important in oncology. Networks that span different disciplines could help to promote interdisciplinary research projects among young scientists and improve exchange in professional practice and education with the implication of improved patient care.

**Supplementary Information:**

The online version contains supplementary material available at 10.1007/s00432-023-04937-2.

## Introduction

Modern, personalized oncological treatment concepts can only be implemented through optimal interdisciplinary and multiprofessional collaboration (Soukup et al. 2018). Interdisciplinary care has been recommended best practice in oncology for more than 25 years and has been adopted by many institutions (Selby et al. 2019). The tumorboard is a prime example for interdisciplinary decision-making of treatment regiments and is a central component of every certified cancer centre. It is seen as indispensable for patient care, and its implementation has been strongly recommended in many guidelines (Homayounfar et al. 2014). The German Cancer Society has proposed a three-stage model for certified institutions treating cancer patients. In this model, organ-specific centres for common tumor types represent a basis for interdisciplinary care. The next stage is the networking oncological centre (Cancer Centre), which pursues the diagnosis and therapy of all tumor entities and promotes interdisciplinary and multiprofessional cooperation between these organ-specific centres. The third level is represented by oncological centres (so-called Comprehensive Cancer Centre, CCC) which integrate oncological translational and clinical research in cancer patients’ care. Such structures require interdisciplinary trained physicians who operate as a team for every individual patient (Güttler et al. 2012). Interdisciplinary care is sometimes used to describe care teams from different professions such as nurses, physical therapists and physicians as well as teams formed by physicians from different specialities (e.g. surgeons, radiologists and medical oncologists).

To reduce ambiguity in this article, we will follow the definition suggested by Laura Petri, who understands interdisciplinary care as “an interpersonal process characterized by healthcare professionals from multiple disciplines with shared objectives, decision-making, responsibility, and power working together to solve patient care problems; the process is best attained through an interprofessional education that promotes an atmosphere of mutual trust and respect” (Petri 2010). We explicitly include all medical professionals directly and indirectly partaking in cancer patient care, including but not limited to nurses, physical therapists, physicist, biologists, scientists geneticist and physicians of all fields.

A recent meta-analysis of studies showed that discussion of oncologic patient cases in an interdisciplinary setting lead to a change in diagnostic procedures in around a third of cases and a change in therapeutical management in over half of cases. However, those studies were heterogeneous and a significant number of the studies reported much smaller proportion of cases with changed diagnostic or therapeutic approach (Pillay et al. 2016). Despite some studies reporting improved patient outcomes including overall survival, significant differences in methodology and cohort characteristics impede the impact quantification of the interdisciplinary care (Liu et al. 2020; Hong et al. 2010; Munro et al. 2015). Another recent meta-analysis reports a positive effect of interdisciplinary tumorboards on 5-year survival, meanwhile only five hereby analyzed studies question the generality of the results for all oncologic entities. "The results of one trial were censored because it "increased heterogeneity of results" by failing to demonstrate a survival benefit, although it met inclusion criteria (Wille-Jørgensen et al. 2013; Algwaiz et al. 2020). Of note, the main research focus on effects of multidisciplinary tumorboards. Although comprehensive studies of the interdisciplinary care following the patient case discussions seems to be hardly quantifiable, it might be the parameter of consequence. Some indirect benefits of cooperation between different healthcare specialties as reduced length of hospital stay or medication use could be demonstrated. Some data insights point toward an increased patient recruitment in clinical studies following case discussion in multidisciplinary tumorboards. Nevertheless, patient-centred outcome analysis is of urgent need. (Walkenhorst, et al. 2015; Kuroki et al. 2010).

In Germany, we are moving toward a competence-oriented learning structure in the medical studies programme, which is currently undergoing a transformation. The planned interlinking of theory and practice as well as the strong focus on higher level competences instead of individual topics is a fundamental step toward an interdisciplinary education.

However, an even greater paucity of data with regard to interdisciplinary training in oncology impedes discussion and improvement of training conditions. A recent study investigating frequency of interdisciplinary training in different oncologic specialties in the US showed that trainees report a significant lower frequency of interdisciplinary training when compared to program directors, and that even according to program directors interdisciplinary education is only part of the curriculum of less than half of surgical or geriatric oncologists (Akthar et al. 2018).

Even though there is a strong goal of alignment in medical education in Germany, the definitions of interdisciplinary medical training in oncology remain unclear. Neither the extent of interdisciplinary training nor the views of the next generation of oncologists regarding interdisciplinarity in clinical care and research are known. Current reports highlight the importance of interdisciplinary research in science education (Daniel et al. 2022).

An example of interdisciplinary cooperation in research are the collaborative structures (Collaborative Research Centres, Research Training Groups) set up by the German Research Foundation, in which young researchers from different disciplines work together on a superordinate goal. Although interdisciplinary cooperation is proven to be powerful, it requires overcoming a number of barriers with the need for constant communication (Haythornthwaite 2006).

The initiative “Young Oncologist United” is a joint effort of junior groups of different German medical societies with a clinical or preclinical oncologic interest to improve interdisciplinary cooperation in patient care, research, and training in oncology (Mäurer et al. 2022). Another important goal is to build and strengthen a community of mutual support, communication, and exchange e.g. through joint meetings and events.

In the following, we present results from a survey on the importance of interdisciplinary oncology care as seen by future oncologists from multiple different specialties.

## Methods

The aim of the online survey was to assess the need for interdisciplinarity at both the educational and research levels, as well as the needs of young oncologists and other disciplines involved in oncology.

The survey language was German and it consisted of both single- and multiple choice questions; six-point-Likert-Scales as well as fill-in responses were utilized (for the complete questionnaire, see Supplemental I. An English translation can be found in Supplemental II) (Likert 1932). The response to all questions was voluntary, anonymous and every question was skippable. After completion of the survey, a computerized matrix was programmed, and the questionnaire was distributed to all participating groups.

The survey was conducted among all junior oncology groups represented in YOU between November 2021 and March 2022. The junior research groups listed in Table 1 participated in the survey. A total of 294 participants completed the questionnaire in full. As the aim of the survey was to investigate opinions on interdisciplinary care in the next generation, answers from respondents holding a professorship (*n* = 4) were excluded.

**Table 1:** Overview of the participating oncology junior research groups

Discipline	Short name	Description
General and visceral surgery	CAJC	Surgical Working Group Young Surgery Young Surgeons Group of the German Society for General and Visceral Surgery (DGAV)
Gastroenterology	AG Junge Gastroenterologie	Task Force Young Gastroenterology, German Society for Gastroenterology, Digestive and Metabolic Diseases (DGVS)
Gynecology	JAGO	Young Academy of Gynecological Oncology
	AGO Young Talents	Project for the promotion of young medical oncologists of the “Arbeitsgemeinschaft Gynäkologische Onkologie” (AGO) of the German Cancer Society (DKG)
Hematology and internal oncology	YMO	Young Medical Oncologists Junior Research Group of the “Arbeitsgemeinschaft Internistische Onkologie” (AIO) of the German Cancer Society (DKG)
	junge DGHO	Junior Research Group of the German Society for Hematology and Medical Oncology (DGHO)
Human genetics	Junge Humangenetik	Junior Research Group of the German Society of Human Genetics (GfH)
Neuro-Oncology	YoungNOA	Junior Research Group of the Neuro-oncological Working Group of the DKG (NOA)
Nuclear medicine	Young DGN	Junior Research Group of the German Society of Nuclear Medicine (DGN)
Palliative medicine	Junge DGP	Junior Group of the German Association for Palliative Medicine (DGP)
Radiotherapy and Radiooncology	junge DEGRO	Junior Research Group of the German Society for Radiooncology (DEGRO)
Thoracic Oncology	YTO	Young Thoracic Oncologists
Urology	GeSRU und GeSRU-Academics	German Society of Residents in Urology Research Group of German Society of Residents in Urology

## Results

### General aspects and demographics

Table 2 summarizes participants’ characteristics and demographics. The median age of the participants was 33.6 years.

**Table 2:** General data and demographics

Parameter	Number (%)
Sex	
Female	159 (54.8%)
Male	131 (45.2%)
Age	
20–25	5 (1.7%)
26–30	77 (26.6%)
31–35	109 (37.6%)
36–40	76 (26.2%)
41–45	18 (6.2%)
45–50	2 (0.7%)
> 50	3 (1.0%)
Highest scientific degree	
None	68 (23.4%)
Doctoral degree	206 (71.0%)
Habilitation	13 (4.5%)
Master of science	2 (0.7%)
Others	1 (0.3%)
Professional background	
Physician	268 (92.4%)
Biologist	11 (3.8%)
Medical physicist	5 (1.7%)
Nurse	2 (0.7%)
Physiotherapist	2 (0.7%)
Psychologist	1 (0.3%)
Social worker	1 (0.3%)
Residency training (*n* = 268)	
1. Year	12 (4.5%)
2. Year	27 (10.1%)
3. Year	27 (10.1%)
4. Year	35 (13.1%)
5. Year	35 (13.1%)
≥ 6. Year	22 (8.2%)
Board certified physician	44 (16.4%)
Attending	66 (24.6%)
Subject	
Internal medicine ± specification	122 (45.5%)
Gynecology	55 (20.5%)
Radiotherapy and radiation-oncology	38 (14.2%)
General or visceral surgery	15 (5.6%)
Human genetics	14 (5.2%)
Neurosurgery	7 (2.6%)
Urology	7 (2.6%)
Neurology	5 (1.9%)
Dermatology	1 (0.3%)
Others	2 (0.7%)

The majority of participants were associated with the profession of medicine (92.2%). The specialty of gastroenterology was most frequently represented (24.5%), followed by gynecology (18.7%).

### Interdisciplinarity in everyday work life

**Fig. 1:**
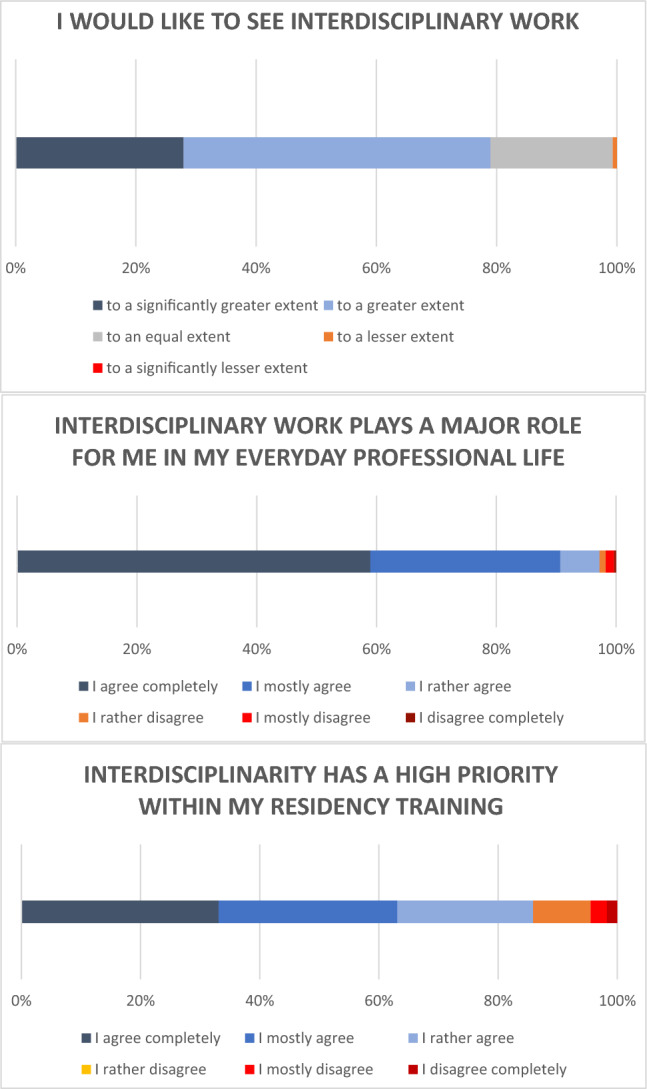
Statements on interdisciplinary work in everyday life

90.7% of the respondents completely or mostly agreed with the statement that interdisciplinary work plays a major role in their daily work (Fig. 1). Most participants even wished for interdisciplinary work to a greater or significantly greater extent (78.9%).

### Interdisciplinarity in education

**Fig. 2:**
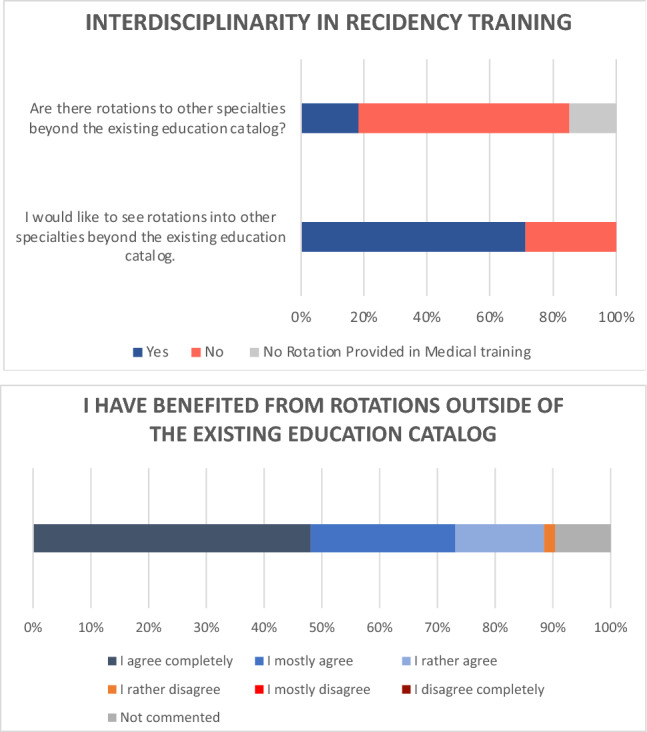
Statements on interdisciplinary education

With regard to residency training, the vast majority assigned a high priority to interdisciplinary training (63.1% completely or mostly agree). Only 53 participants (18.3%) had the opportunity to take planned rotations to other specialties beyond the continuing education catalog. For 43 respondents (14.8%), rotations are not part of residency training. Yet, our data indicated a high interest in other specialties (Fig. 2). 207 participants would like to participate in rotations to other specialties (71.4%). The majority of those who completed rotations stated that they had benefited mostly or greatly from them (73.1%).

### Interdisciplinary in research

**Fig. 3:**
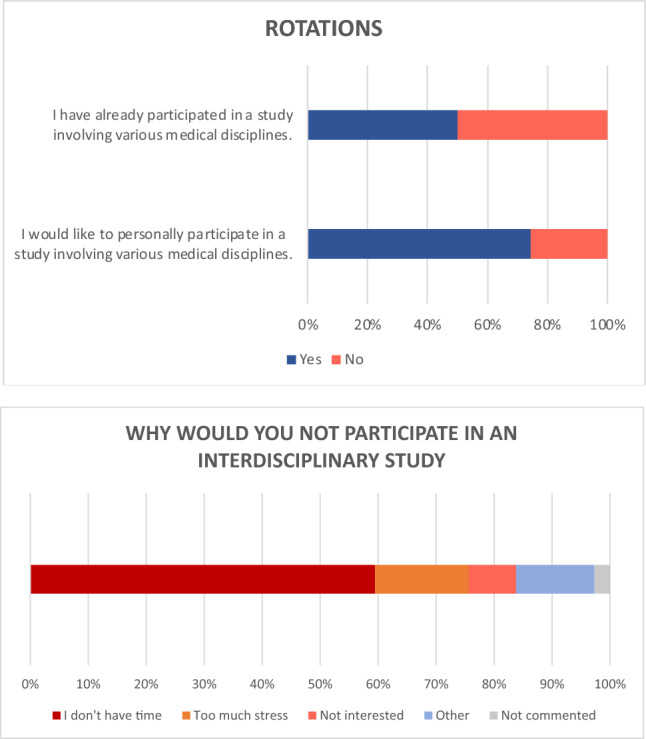
Statements on interdisciplinary research

About half of the respondents (50.3%) reported, having participated in a scientific study involving various oncology disciplines. Of those who had not yet been involved in interdisciplinary research, 80.1% indicated an interest in participating in a multidisciplinary/interdisciplinary study project in the future (Fig. 3).

**Fig. 4:**
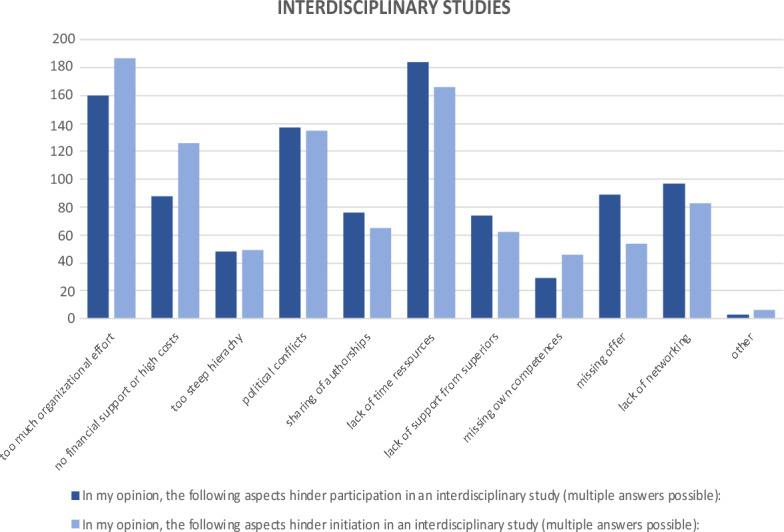
Statements on interdisciplinary research

High organizational costs, lack of time resources, and possible political conflicts were cited as reasons against participating in or initiating an interdisciplinary study (Fig. 4).

### Interdisciplinary networks

**Fig. 5:**
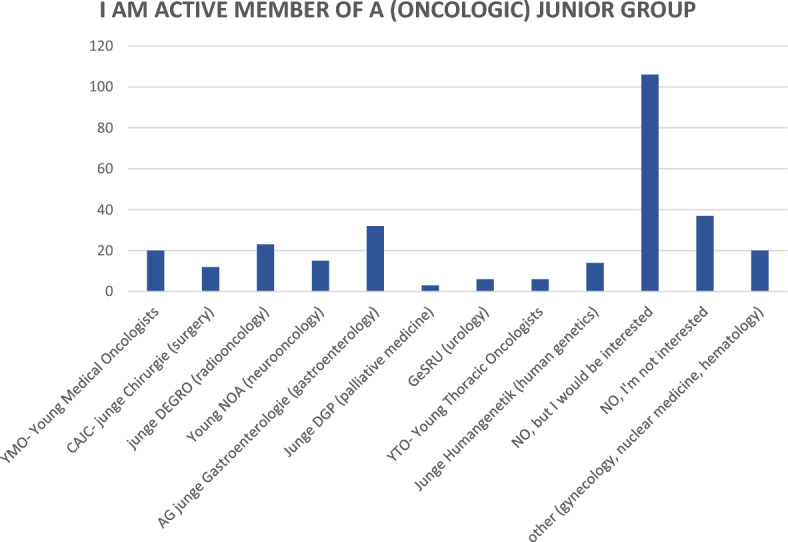
Bar chart on activity in junior groups

Of the 294 respondents, 151 (51.4%) indicated that they were active in an oncological junior group (Fig. 5). Of the 143 previously inactive participants, 106 (74.1%) again indicated that they would be interested in joining the group. Only 37 participants declined to actively participate.

**Fig. 6:**
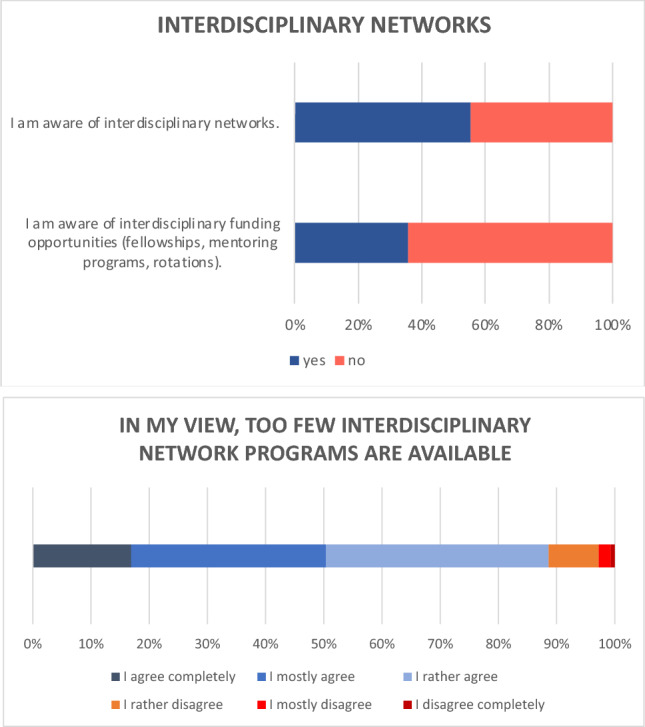
Bar chart on activity in junior groups

161 of the 290 participants (55.5%) indicated that they were already aware of interdisciplinary networks. 104 (35.9%) were aware of interdisciplinary funding opportunities (Fig. 6).

## Discussion

We here present the first survey on opinions on interdisciplinary care and research among junior oncologists in Germany. Our survey results underscore the perception of the importance of interdisciplinary collaboration in modern oncology. The majority of respondents welcomed the already existing interdisciplinary cooperation in the areas of daily work, education and research. Most respondents wished for even stronger collaboration and training.

However, only half of the respondents have actively participated in interdisciplinary oncology study activities so far, more than 70% of the non-participants would like to do so. Participants believe interdisciplinarity is important and would welcome expansion of existing support programs.

Interdisciplinary work has become indispensable in oncology and is a substantial guarantor of quality in cancer care practices (Tremblay et al. 2017; Velde et al. 2014; Jemal et al. 2011). In fact, oncology has been a driver in institutionalized collaboration in the medical field, as evidenced by the early establishment of regularly scheduled interdisciplinary expert conferences (“tumorboard review”) and a well-regulated certification process of interdisciplinary cancer centers (Hawk and Viner 2006; Kowalski et al. 2017; Wallwiener et al. 2012; Henson et al. 1990). Furthermore, scientific progress in oncology heavily benefits from interdisciplinary cooperation, also on an international level of collaboration (Pui et al. 2015; Gaspar et al. 2015; Jaffee et al. 2017). As the population is aging, an increasing demand of interdisciplinary care is expected due to a growing number of oncologic patients (Sung et al. 2021; Terret et al. 2007). Simultaneously interdisciplinarity is highly valued in the management of pediatric oncologic patients (Wein et al. 2010).

Cancer medicine is developing rapidly and cancer is increasingly becoming a chronic disease. The constant improvements in the management of acute complications make it more and more possible to treat elderly and multimorbid patients with intensive oncological treatment strategies. As well as the path to treatment, the increasingly complex therapies with a broader spectrum of adverse effects and more nuanced approaches to treating these side effects require intensive interdisciplinary cooperation (Bergwelt-Baildon et al. 2010; Schellongowski and Staudinger 2012).

A catalyst for interdisciplinary collaboration in oncology is the increasing complexity of diagnostic and therapeutic options (Meric-Bernstam et al. 2021; Lambin et al. 2017). For instance, the increasing use of next-generation sequencing (NGS) for patients with metastatic cancers and the use of DNA methylation profiles demand high expertise in data interpretation and a meticulous evaluation of potential clinical consequences and have, therefore, led to the implementation of specialized interdisciplinary molecular tumorboards (Mosele et al. 2020; Capper et al. 2018; Heinrich et al. 2022).

Although interdisciplinary patient care is now part of everyday clinical practice, studies on multidisciplinary teams and on an improvement of patient outcome through multidisciplinary teams have shown nonuniform results. However, negative results were probably mostly related to shortcomings of the chosen study design and the majority of studies rather suggest a benefit of the implementation of interdisciplinary tumorboards on patient management, also regarding hard outcome parameters such as overall survival (Pillay et al. 2016; Wille-Jørgensen et al. 2013; Lamb et al. 2011; Forrest et al. 2005; Freeman, et al. 2011; Freeman, et al. 2010; Brännström et al. 2015; Davies et al. 2006). For instance, in a study evaluating differences in overall survival in patients with Hepatocellular Carcinoma (HCC) before and after the establishment of a Multidisciplinary Clinic (MDC) for HCC, patients diagnosed after the MDC initiation had a median survival of 13.2 months compared to only 4.8 months in patients diagnosed before the MDC initiation (*p* = 0.005). In the multivariate analysis, being seen in the MDC was independently associated with improved overall survival after adjusting for tumor stage and reception of curative treatment regimen (hazard ratio 2.5, 95% confidence interval 2–3) (Yopp et al. 2014). As interdisciplinarity is considered the standard of care, it is becoming more and more difficult to deny patient interdisciplinary care in order to assess its impact on patient’s outcome.

About half of the respondents to this survey indicated that they had already participated in a study involving different oncology disciplines. Of those who had not yet been involved in interdisciplinary research, 80% indicated an interest. The main obstacles reported are lack of time, high organizational costs and political conflicts. Importantly, only few clinical trials are non-interdisciplinary as modern cancer medicine demands involvement of various medical specialties and allied healthcare professionals. Even study designing demands collaboration with other researchers and representatives from fields such as biostatistics, clinical trials management or regulatory bodies. The GTCSG (German Testicular Cancer Study Group) is a successful example of an interdisciplinary research collaborative, which keeps a low threshold for interested young colleagues in training. The results of our survey reveal that there remain many organizational barriers to interdisciplinary collaboration. These barriers can be avoided by working in a multi-institutional setting as recently shown (Brown et al. 2022).

This work is subject to certain limitations. The survey was primarily completed by physicians in advanced training, i.e., physicians who should have a deeper overview of interdisciplinary work. In addition, there may have been increased participation in the survey by individuals who are fundamentally favorable toward interdisciplinary collaboration and research (selection bias).

Participants were largely physicians in internal medicine, followed by gynecologists, radiation oncologists, and a large mixed remainder. However, we feel that this may well present the reality of oncological patient care and academia. Furthermore, this study primarily represents views on interdisciplinarity in the setting of the German health system and the transferability to other medical systems remains unclear.

The results generated by our survey highlight the wish for structured training programs that embrace working in interdisciplinary cancer teams. As most obstacles are organizational, these will need to be addressed from higher institutional hierarchies (Schafer 2010). Importantly, our study indicates that the long lamented lack of physician scientists is not due to a lack of enthusiasm from the next generation but perceived organizational hurdles. One way to approach the lack of time for research could be by strengthening clinician scientist programs (Richter-Kuhlmann 2023). Acknowledging the various gains, the additional workload of multidisciplinary teams should be compensated by appropriate remuneration (Winters et al. 2021). Medical students should concentrate at an early stage on consultation occasions that go beyond the pure acquisition of skills and knowledge, irrespective of the specialty. This basic training could provide a future foundation of interdisciplinary oncology education (Raes, et al. 2014; Wissing 2018).

## Conclusion

Improved and expanded interdisciplinary collaboration is challenging but essential for the future of oncology. While interdisciplinary work already plays an important role for most survey participants interested in oncology, the majority would like to see even more interdisciplinary networking regarding daily clinical work and education. Although the interest in interdisciplinary research and network is high among the survey participants, various factors could be identified that make practical implementation difficult. Lack of time resources, excessive organizational effort, and possible political conflicts between institutions are the most frequently cited obstacles to successful interdisciplinary research projects. Likewise, there is no interdisciplinary organizational structure at the junior oncology level. Despite methodological weaknesses (selection bias), the survey provides valuable information on the importance of interdisciplinarity among young oncologists. Multi-professional and interdisciplinary networks could help to promote interdisciplinary research projects among young scientists and improve interdisciplinary exchange in professional practice, training and education.

## Supplementary Information

Below is the link to the electronic supplementary material.

Supplementary file1 (DOCX 24 KB)

Supplementary file2 (DOCX 20 KB)

## Data Availability

The data sets created and/or analyzed as part of the study can be obtained from the author upon reasonable request.

## References

[CR1] Akthar AS (2018). Interdisciplinary oncology education: a national survey of trainees and program directors in the United States. J Cancer Educ.

[CR2] Algwaiz G (2020). Do multidisciplinary tumor board discussions correlate with increase in 5-year survival? A meta-analysis study. Global Journal on Quality and Safety in Healthcare.

[CR3] Brännström F (2015). Multidisciplinary team conferences promote treatment according to guidelines in rectal cancer. Acta Oncol.

[CR4] Brown S-A (2022). Establishing an interdisciplinary research team for cardio-oncology artificial intelligence informatics precision and health equity. Am Heart J plus.

[CR5] Capper D (2018). DNA methylation-based classification of central nervous system tumours. Nature.

[CR6] Daniel KL (2022). Challenges facing interdisciplinary researchers: findings from a professional development workshop. PLoS ONE.

[CR7] Davies AR (2006). The multidisciplinary team meeting improves staging accuracy and treatment selection for gastro-esophageal cancer. Dis Esophagus.

[CR8] Forrest LM (2005). An evaluation of the impact of a multidisciplinary team, in a single centre, on treatment and survival in patients with inoperable non-small-cell lung cancer. Br J Cancer.

[CR9] Freeman RK (2010). The effect of a multidisciplinary thoracic malignancy conference on the treatment of patients with lung cancer. Eur J Cardiothorac Surg.

[CR10] Freeman RK (2011). The effect of a multidisciplinary thoracic malignancy conference on the treatment of patients with esophageal cancer. Ann Thorac Surg.

[CR11] Gaspar N (2015). Ewing sarcoma: current management and future approaches through collaboration. J Clin Oncol.

[CR12] Güttler F (2012). Interdisziplinäre Tumorkonferenzen - Regionale und überregionale telemedizinische und teleradiologische Anbindung von Tumorzentren. Onkologe.

[CR13] Hawk E, Viner JL (2006). What is the future of oncology? National cancer institute initiatives to improve research, development, and implementation in cancer prevention and treatment. Semin Oncol.

[CR14] Haythornthwaite C et al (2006) Challenges for research and practice in distributed, interdisciplinary collaboration. In: Hine C (ed) New infrastructures for knowledge production: understanding e-science. Hershey, IGI Global, p 143–166

[CR15] Heinrich K (2022). Lessons learned: the first consecutive 1000 patients of the CCCMunich^LMU^ molecular tumor board. J Cancer Res Clin Oncol.

[CR16] Henson DE (1990). Results of a national survey of characteristics of hospital tumor conferences. Surg Gynecol Obstet.

[CR17] Homayounfar K, Lordick F, Ghadimi M (2014). Qualitätssicherung: Multidisziplinäre Tumorboards—trotz Problemen unverzichtbar. Dtsch Arztebl International.

[CR18] Hong NJL (2010). Examining the potential relationship between multidisciplinary cancer care and patient survival: An international literature review. J Surg Oncol.

[CR19] Jaffee EM (2017). Future cancer research priorities in the USA: a Lancet oncology commission. Lancet Oncol.

[CR20] Jemal A (2011). Global cancer statistics. CA Cancer J Clin.

[CR21] Kowalski C (2017). Shifting cancer care towards multidisciplinarity: the cancer center certification program of the German cancer society. BMC Cancer.

[CR22] Kuroki L (2010). Addressing clinical trials: can the multidisciplinary tumor board improve participation? A study from an academic women's cancer program. Gynecol Oncol.

[CR23] Lamb BW (2011). Quality of care management decisions by multidisciplinary cancer teams: a systematic review. Ann Surg Oncol.

[CR24] Lambin P (2017). Radiomics: the bridge between medical imaging and personalized medicine. Nat Rev Clin Oncol.

[CR25] Likert R (1932). A technique for the measurement of attitudes. Archives of Psychology.

[CR26] Liu JC (2020). The impact of the multidisciplinary tumor board on head and neck cancer outcomes. Laryngoscope.

[CR27] Mäurer MA (2022). Erstmalige interdisziplinäre DKK-Programmplanung durch Zusammenschluss onkologischer Nachwuchsgruppen. Forum.

[CR28] Meric-Bernstam F (2021). Enhancing anti-tumour efficacy with immunotherapy combinations. Lancet.

[CR29] Mosele F (2020). Recommendations for the use of next-generation sequencing (NGS) for patients with metastatic cancers: a report from the ESMO precision medicine working group. Ann Oncol.

[CR30] Munro A (2015). Do multidisciplinary team (MDT) processes influence survival in patients with colorectal cancer? A population-based experience. BMC Cancer.

[CR31] Petri L (2010). Concept analysis of interdisciplinary collaboration. Nurs Forum.

[CR32] Pillay B (2016). The impact of multidisciplinary team meetings on patient assessment, management and outcomes in oncology settings: a systematic review of the literature. Cancer Treat Rev.

[CR33] Pui C-H (2015). Childhood acute lymphoblastic leukemia: progress through collaboration. J Clin Oncol.

[CR34] Raes P (2014). The active participation of German-speaking countries in conferences of the association for medical education in Europe (AMEE) between 2005 and 2013: a reflection of the development of medical education research?. GMS Z Med Ausbild.

[CR35] Richter-Kuhlmann E (2023). Clinician scientists: Synthese von Klinik und Forschung. Dtsch Arztebl International.

[CR36] Schafer AI (2010). The vanishing physician-scientist?. Transl Res.

[CR37] Schellongowski P, Staudinger T (2012). Intensivmedizinische Probleme des hämatoonkologischen Patienten. Medizinische Klinik - Intensivmedizin Und Notfallmedizin.

[CR38] Selby P (2019). The value and future developments of multidisciplinary team cancer care. Am Soc Clin Oncol Educ Book.

[CR39] Soukup T (2018). Successful strategies in implementing a multidisciplinary team working in the care of patients with cancer: an overview and synthesis of the available literature. J Multidiscip Healthc.

[CR40] Sung H (2021). Global cancer statistics 2020: GLOBOCAN estimates of incidence and mortality worldwide for 36 cancers in 185 countries. CA Cancer J Clin.

[CR41] Terret C (2007). Multidisciplinary approach to the geriatric oncology patient. J Clin Oncol.

[CR42] Tremblay D (2017). Effects of interdisciplinary teamwork on patient-reported experience of cancer care. BMC Health Serv Res.

[CR43] van de Velde CJ (2014). EURECCA colorectal: multidisciplinary management: European consensus conference colon & rectum. Eur J Cancer.

[CR44] von Bergwelt-Baildon M (2010). CCC meets ICU: redefining the role of critical care of cancer patients. BMC Cancer.

[CR45] Walkenhorst U (2015). Position statement GMA committee—“interprofessional education for the health care professions”. GMS Zeitschrift Fur Medizinische Ausbildung.

[CR46] Wallwiener M (2012). Multidisciplinary breast centres in Germany: a review and update of quality assurance through benchmarking and certification. Arch Gynecol Obstet.

[CR47] Wein S, Pery S, Zer A (2010). Role of palliative care in adolescent and young adult oncology. J Clin Oncol.

[CR48] Wille-Jørgensen P (2013). Result of the implementation of multidisciplinary teams in rectal cancer. Colorectal Dis.

[CR49] Winters DA (2021). The cancer multidisciplinary team meeting: in need of change? History, challenges and future perspectives. BJU Int.

[CR50] Wissing F (2018). Nationaler Kompetenzbasierter Lernzielkatalog Medizin und Zahnmedizin (NKLM/NKLZ). Bundesgesundheitsblatt - Gesundheitsforschung - Gesundheitsschutz.

[CR51] Yopp AC (2014). Establishment of a multidisciplinary hepatocellular carcinoma clinic is associated with improved clinical outcome. Ann Surg Oncol.

