# Supplementary material for: Importance of interdisciplinarity in modern oncology: results of a national intergroup survey of the Young Oncologists United (YOU)
Source: J Cancer Res Clin Oncol. 2023 Jun 1;149(12):10075–84. doi: 10.1007/s00432-023-04937-2 (PMC10423150; doi:10.1007/s00432-023-04937-2)
Supplement: Supplementary file 1 — Supplementary file1 (DOCX 24 KB) [file 432_2023_4937_MOESM1_ESM.docx]

**Supplemental I - Fragebogen**

Liebe KollegInnen,

wir begrüßen Sie herzlich zu einer Umfrage unter jungen onkologisch tätigen KollegInnen.
Zur Förderung der Fächer- und Professionen-übergreifenden Netzwerke und Weiterbildung möchten wir Sie gerne anonym zu Ihrer Einstellung hinsichtlich einer interdisziplinären und multiprofessionellen Zusammenarbeit im Hinblick auf Weiterbildung und Forschung befragen.

Diese Umfrage umfasst 5 Abschnitte: Allgemeine Fragen, Interdisziplinäres Arbeiten und Interdisziplinäre Forschung, Multiprofessionelles Arbeit und Multiprofessionelle Forschung.
Der Zeitaufwand beträgt voraussichtlich weniger als 10 Minuten.

Wir würden uns daher sehr über Ihre Teilnahme freuen.

Wir starten nun mit allgemeinen Fragen zu Ihrer Person.

| **Frage** | **Antwortmöglichkeiten** |
| --- | --- |
| Alter | Zahl |
| Gender | Mann  Frau  Divers |
| Höchster wissenschaftlicher Grad | keiner  Promotion  Habilitation  Professur  Sonstiges |
| Berufsgruppenzugehörigkeit | Medizin  Pflege  Psychologie / Psychoonkologie  Biologie  Soziale Arbeit  Therapieberufe (Physiotherapie / Logopädie / Ergotherapie)  Seelsorge  Sonstiges |
| Weiterbildungsstand / Position | Dropdown:  1. Ausbildungsjahr  2. Ausbildungsjahr  3. Ausbildungsjahr  4. Ausbildungsjahr  5. Ausbildungsjahr  ≥ 6. Ausbildungsjahr  Facharzt  Oberarzt |
| Facharztausbildung / Facharzt | Innere Medizin und Hämatologie und Onkologie  Allgemein- oder Viszeralchirurgie  Strahlentherapie & Radioonkologie  Neurologie  Neurochirurgie  Gynäkologie  Gastroenterologie  Dermatologie  Urologie  HNO  Radiologie  Humangenetik  Sonstige:: Freitext |
| Ich bin Mitglied in den folgenden Fachgesellschaften | Dropdown (Mehrfachwahl):  1.  DKG - AIO  DKG - ACO  DKG - AGO  DKG - ARO  DKG - NOA  2.  DEGRO  DGCH  DGAV  DGHO  DGGG  DGN  DGNC  DGVS  DGP (Palliativmedizin)  Sonstige:  keine |
| Ich bin aktives Mitglied einer (onkologischen) Nachwuchsgruppe | ja (Dropdown: YMO, junge DGHO, CAJC, junge DEGRO, Young NOA, AG junge Gastroenterologie, Junge DGP, Sonstige: Freitext)  nein, ich hätte aber Interesse mitzumachen  nein, Begründung Dropdown:  ich weiß nicht was Mitglieder von Nachwuchsgruppen tun  keine Zeit  kein Interesse  zu viel Stress im Arbeitsalltag  ich fühle mich nicht kompetent genug  ich konzentriere mich lieber auf die Aus- / Weiterbildung  erscheint mir nicht wichtig  Abneigung gegenüber Gruppenmitgliedern  Freitext: |
| Ich bin über die Arbeit meiner fachspezifischen Nachwuchsgruppe gut informiert | stimme ich vollkommen zu  stimme ich überwiegend zu  stimme ich eher zu  stimme ich eher nicht zu  stimme ich größtenteils nicht zu  stimme ich überhaupt nicht zu |
| **Interdisziplinäres Arbeiten** | |
| Interdisziplinäres Arbeiten spielt für mich im Berufsalltag eine große Rolle | stimme ich vollkommen zu  stimme ich überwiegend zu  stimme ich eher zu  stimme ich eher nicht zu  stimme ich größtenteils nicht zu  stimme ich überhaupt nicht zu |
| Ich wünsche mir interdisziplinäres Arbeiten | in einem deutlich größeren Ausmaß  in einem größeren Ausmaß  in einem gleichen Ausmaß  in einem geringeren Ausmaß  in einem deutlich geringeren Ausmaß |
| Interdisziplinarität hat einen hohen Stellenwert innerhalb meiner Facharztweiterbildung | stimme ich vollkommen zu  stimme ich überwiegend zu  stimme ich eher zu  stimme ich eher nicht zu  stimme ich größtenteils nicht zu  stimme ich überhaupt nicht zu |
| Finden über den Weiterbildungskatalog hinaus Rotationen in andere Fachgebiete statt? | ja  nein  wenn ja:  Ich habe von Rotationen außerhalb des Weiterbildungskataloges profitiert:  stimme ich vollkommen zu  stimme ich überwiegend zu  stimme ich eher zu  stimme ich eher nicht zu  stimme ich größtenteils nicht zu  stimme ich überhaupt nicht zu |
| Ich wünsche mir Rotationen in andere Fachgebiete über den WB-Katalog hinaus | ja  nein |
| **Interdisziplinäre Forschung** | |
| Ich habe mich bereits an einer Studie unter Mitwirkung verschiedener medizinischer Disziplinen beteiligt? | ja  nein  wenn letzte Frage nein, dann:  Ich würde mich persönlich gern an einer Studie unter Mitwirkung verschiedener medizinischer Disziplinen beteiligen.  ja  nein,  erscheint mir nicht wichtig  bringt mir persönlich nichts  kein Interesse  keine Zeit  zu viel Stress  Freitext: |
| Folgende Aspekte behindern meiner Meinung nach die Beteiligung an einer interdisziplinären Studie:  max 5 Nennungen | (Mehrfachnennung möglich)  Zu hoher Organisatorischer Aufwand  Keine finanzielle Unterstützung/zu hohe Kosten  Zu steile Hierarchie  Politische Konflikte (z.B. mit anderen Abteilungen)  Teilung der Autorenschaften  Mangelnde zeitliche Ressourcen  Fehlende Unterstützung durch Vorgesetzte  Fehlende eigene Kompetenzen  Fehlendes Angebot  Fehlende Vernetzung  Freitext: |
| Folgende Aspekte behindern meiner Meinung die Initiierung einer interdisziplinären Studie?  max 5 Nennungen | (Mehrfachnennung möglich)  Zu hoher Organisatorischer Aufwand  Keine finanzielle Unterstützung/zu hohe Kosten  Zu steile Hierarchie  Politische Konflikte (z.B. mit anderen Abteilungen)  Teilung der Autorenschaften  Mangelnde zeitliche Ressourcen  Fehlende Unterstützung durch Vorgesetzte  Fehlende eigene Kompetenzen  Fehlendes Angebot  Fehlende Vernetzung  Freitext: |
| Mir sind interdisziplinäre Netzwerke bekannt. | ja  nein |
| Ich kenne interdisziplinäre Fördermöglichkeiten (Stipendien, Mentoringprogramme, Rotationen)? | ja  nein |
| Aus meiner Sicht sind zu wenig interdisziplinäre Netzwerkprogramme verfügbar | stimme ich vollkommen zu  stimme ich überwiegend zu  stimme ich eher zu  stimme ich eher nicht zu  stimme ich größtenteils nicht zu  stimme ich überhaupt nicht zu |
| **Multiprofessionelle Zusammenarbeit** | |
| Multiprofessionelle Zusammenarbeit spielt für mich im Berufsalltag eine große Rolle | stimme ich vollkommen zu  stimme ich überwiegend zu  stimme ich eher zu  stimme ich eher nicht zu  stimme ich größtenteils nicht zu  stimme ich überhaupt nicht zu |
| Ich wünsche mir multiprofessionelle Zusammenarbeit | in einem deutlich größeren Ausmaß  in einem größeren Ausmaß  in einem gleichen Ausmaß  in einem geringeren Ausmaß  in einem deutlich geringeren Ausmaß |
| Für Ärzt:innen  Multiprofessionalität hat einen hohen Stellenwert innerhalb meiner Facharztweiterbildung | stimme ich vollkommen zu  stimme ich überwiegend zu  stimme ich eher zu  stimme ich eher nicht zu  stimme ich größtenteils nicht zu  stimme ich überhaupt nicht zu |
| Für Ärzt:innen  Finden über den Weiterbildungskatalog hinaus Rotationen in andere Fachgebiete statt? | ja  nein |
| wenn letzte Frage ja:  Ich habe von Rotationen außerhalb des Weiterbildungskataloges profitiert. | stimme ich vollkommen zu  stimme ich überwiegend zu  stimme ich eher zu  stimme ich eher nicht zu  stimme ich größtenteils nicht zu  stimme ich überhaupt nicht zu |
| Ich wünsche mir Rotationen in andere Fachgebiete über den WB-Katalog hinaus | ja  nein |
| **Multiprofessionelle Forschung** | |
| Ich habe mich bereits an einer Studie unter Mitwirkung verschiedener Berufsgruppen beteiligt? | ja  nein |
| wenn letzte Frage nein, dann:  Ich würde mich persönlich gern an einer Studie unter Mitwirkung verschiedener Berufsgruppen beteiligen. | ja  nein,  erscheint mir nicht wichtig  bringt mir persönlich nichts  kein Interesse  keine Zeit  zu viel Stress  Freitext: |
| Folgende Aspekte behindern meiner Meinung nach die Beteiligung an einer multiprofessionellen Studie:  max 5 Nennungen | (Mehrfachnennung möglich)  Zu hoher Organisatorischer Aufwand  Keine finanzielle Unterstützung/zu hohe Kosten  Zu steile Hierarchie  Politische Konflikte (z.B. mit anderen Abteilungen)  Teilung der Autorenschaften  Mangelnde zeitliche Ressourcen  Fehlende Unterstützung durch Vorgesetzte  Fehlende eigene Kompetenzen  Fehlendes Angebot  Fehlende Vernetzung  Freitext: |
| Folgende Aspekte behindern meiner Meinung die Initiierung einer multiprofessionellen Studie  max 5 Nennungen | (Mehrfachnennung möglich)  Zu hoher Organisatorischer Aufwand  Keine finanzielle Unterstützung/zu hohe Kosten  Zu steile Hierarchie  Politische Konflikte (z.B. mit anderen Abteilungen)  Teilung der Autorenschaften  Mangelnde zeitliche Ressourcen  Fehlende Unterstützung durch Vorgesetzte  Fehlende eigene Kompetenzen  Fehlendes Angebot  Fehlende Vernetzung  Freitext: |
| Mir sind multiprofessionelle Netzwerke bekannt. | ja  nein |
| Ich kenne multiprofessionelle Fördermöglichkeiten (Stipendien, Mentoringprogramme, Rotationen)? | ja  nein |
| Aus meiner Sicht sind zu wenig multiprofessionelle Netzwerkprogramme verfügbar | stimme ich vollkommen zu  stimme ich überwiegend zu  stimme ich eher zu  stimme ich eher nicht zu  stimme ich größtenteils nicht zu  stimme ich überhaupt nicht zu |
