# Supplementary material for: Importance of interdisciplinarity in modern oncology: results of a national intergroup survey of the Young Oncologists United (YOU)
Source: J Cancer Res Clin Oncol. 2023 Jun 1;149(12):10075–84. doi: 10.1007/s00432-023-04937-2 (PMC10423150; doi:10.1007/s00432-023-04937-2)
Supplement: Supplementary file 2 — Supplementary file2 (DOCX 20 KB) [file 432_2023_4937_MOESM2_ESM.docx]

**Supplemental I - Questionnaire**

Dear colleagues,

 We would like to welcome you to a survey among young colleagues
working in oncology.
 In order to promote interdisciplinary and interprofessional networks and continuing education, we would like to anonymously survey you about your attitudes regarding interdisciplinary and multiprofessional collaboration with regard to continuing education and research.

 This survey includes 5 sections: General Questions, Interdisciplinary Working and Interdisciplinary Research, Multiprofessional Working, and Multiprofe ssional Research.
 The time required is expected to be less than 10 minutes.

 We would therefore very much appreciate your participation.

 We will now start with general questions about yourself.

| **Question** | **Answer options** |
| --- | --- |
| Age | Number |
| Gender | Male  Female  Divers |
| Highest scientific degree | none  Promotion  Habilitation  Professorship  Other |
| Professional group affiliation | Medicine  Care  Psychology / Psychooncology  Biology  Social work  Therapy professions (physiotherapy / speech therapy / occupational therapy)  Pastoral care  Other |
| Further training status / position | Dropdown:  1st year of training  2nd year of training  3rd year of training  4th year of training  5th year of training  ≥ 6th year of training  Specialist  Senior physician |
| Residency / Specialist | Internal Medicine and Hematology and Oncology  General or visceral surgery  Radiation Therapy & Radiation Oncology  Neurology  Neurosurgery  Gynecology  Gastroenterology  Dermatology  Urology  ENT  Radiology  Human Genetics  Other:: Free text |
| I am a member of the following professional societies | Dropdown (multiple choice):  1.  DKG - AIO  DKG - ACO  DKG - AGO  DKG - ARO  DKG - NOA  2.  DEGRO  DGCH  DGAV  DGHO  DGGG  DGN  DGNC  DGVS  DGP (Palliative Medicine)  Other:  none |
| I am an active member of a (oncological) junior research group | yes (dropdown: YMO, young DGHO, CAJC, young DEGRO, young NOA, AG junge Gastroenterologie, young DGP, other: free text)  no, but I would be interested to participate  no, reason dropdown:  I do not know what members of junior research groups do  no time  No interest  too much stress in everyday work  I do not feel competent enough  I prefer to concentrate on training / further education  does not seem important to me  Dislike of group members  Free text: |
| I am well informed about the work of my subject-specific junior research group | I agree completely  I agree for the most part  I rather agree  I rather do not agree  I do not agree for the most part  I do not agree at all |
| **Interdisciplinary work** | |
| Interdisciplinary work plays a major role for me in my everyday professional life | I agree completely  I agree for the most part  I rather agree  I rather do not agree  I do not agree for the most part  I do not agree at all |
| I would like to see interdisciplinary work | to a significantly greater extent  to a greater extent  to an equal extent  to a lesser extent  to a significantly lesser extent |
| Interdisciplinarity has a high value within my residency training | I agree completely  I agree for the most part  I rather agree  I rather do not agree  I do not agree for the most part  I do not agree at all |
| Are there rotations to other specialties beyond the continuing education catalog? | yes  no  if so:  I have benefited from rotations outside of the continuing education catalog:  I agree completely  I agree for the most part  I rather agree  I rather do not agree  I do not agree for the most part  I do not agree at all |
| I would like to see rotations into other specialties beyond the WB catalog | yes  no |
| **Interdisciplinary research** | |
| I have already participated in a study involving various medical disciplines? | yes  no  if last question no, then:  I would personally like to participate in a study involving various medical disciplines.  yes  no,  does not seem important to me  brings me personally nothing  No interest  no time  Too much stress  Free text: |
| In my opinion, the following aspects hinder participation in an interdisciplinary study:  max 5 entries | (multiple answers possible)  Too much organizational effort  No financial support/too high costs  Too steep hierarchy  Political conflicts (e.g. with other departments)  Sharing of authorships  Lack of time resources  Lack of support from superiors  Lack of own competences  Missing offer  Lack of networking  Free text: |
| In my opinion, the following aspects hinder the initiation of an interdisciplinary study?  max 5 entries | (multiple answers possible)  Too much organizational effort  No financial support/too high costs  Too steep hierarchy  Political conflicts (e.g. with other departments)  Sharing of authorships  Lack of time resources  Lack of support from superiors  Lack of own competences  Missing offer  Lack of networking  Free text: |
| I am aware of interdisciplinary networks. | yes  no |
| I am aware of interdisciplinary funding opportunities (fellowships, mentoring programs, rotations)? | yes  no |
| In my view, too few interdisciplinary network programs are available | I agree completely  I agree for the most part  I rather agree  I rather do not agree  I do not agree for the most part  I do not agree at all |
| **Multiprofessional cooperation** | |
| Multi-professional collaboration plays a major role for me in my day-to-day professional life | I agree completely  I agree for the most part  I rather agree  I rather do not agree  I do not agree for the most part  I do not agree at all |
| I would like to see multiprofessional cooperation | to a significantly greater extent  to a greater extent  to an equal extent  to a lesser extent  to a significantly lesser extent |
| For doctors  Multi-professionalism has a high priority within my residency training | I agree completely  I agree for the most part  I rather agree  I rather do not agree  I do not agree for the most part  I do not agree at all |
| For doctors  Are there rotations to other specialties beyond the continuing education catalog? | yes  no |
| if last question yes:  I have benefited from rotations outside of the continuing education catalog. | I agree completely  I agree for the most part  I rather agree  I rather do not agree  I do not agree for the most part  I do not agree at all |
| I would like to see rotations into other specialties beyond the WB catalog | yes  no |
| **Multiprofessional research** | |
| I have already participated in a study involving various professional groups? | yes  no |
| if last question no, then:  I would personally like to participate in a study with the participation of various professional groups. | yes  no,  does not seem important to me  brings me personally nothing  No interest  no time  Too much stress  Free text: |
| In my opinion, the following aspects hinder participation in a multiprofessional study:  max 5 entries | (multiple answers possible)  Too much organizational effort  No financial support/too high costs  Too steep hierarchy  Political conflicts (e.g. with other departments)  Sharing of authorships  Lack of time resources  Lack of support from superiors  Lack of own competences  Missing offer  Lack of networking  Free text: |
| In my opinion, the following aspects hinder the initiation of a multiprofessional study.  max 5 entries | (multiple answers possible)  Too much organizational effort  No financial support/too high costs  Too steep hierarchy  Political conflicts (e.g. with other departments)  Sharing of authorships  Lack of time resources  Lack of support from superiors  Lack of own competences  Missing offer  Lack of networking  Free text: |
| I am aware of multiprofessional networks. | yes  no |
| I am aware of multiprofessional funding opportunities (grants, mentoring programs, rotations)? | yes  no |
| From my point of view, too few multiprofessional network programs are available | I agree completely  I agree for the most part  I rather agree  I rather do not agree  I do not agree for the most part  I do not agree at all |
